# Supplementary figures and images for: Sporothrix globosa melanin regulates autophagy via the TLR2 signaling pathway in THP-1 macrophages
Source: PLoS Negl Trop Dis. 2023 May 4;17(5):e0011281. doi: 10.1371/journal.pntd.0011281 (PMC10198546; doi:10.1371/journal.pntd.0011281)

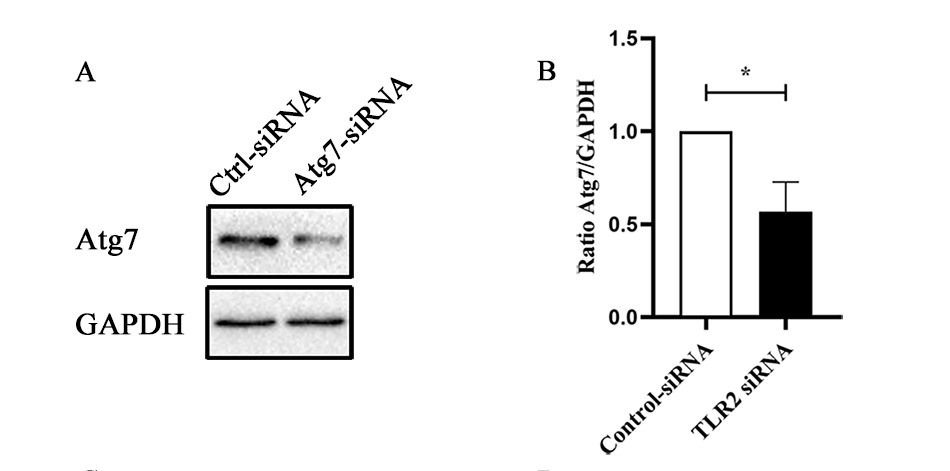

Supplement: S1 Fig — (A) Western blotting of Atg7 was conducted in THP-1 macrophages with or without knockdown treatment. (B) The ratio of Atg7/GAPDH was calculated. *P < 0.05, for the unpaired t-test. Representative images are displayed. (TIF) [file pntd.0011281.s001.tif]

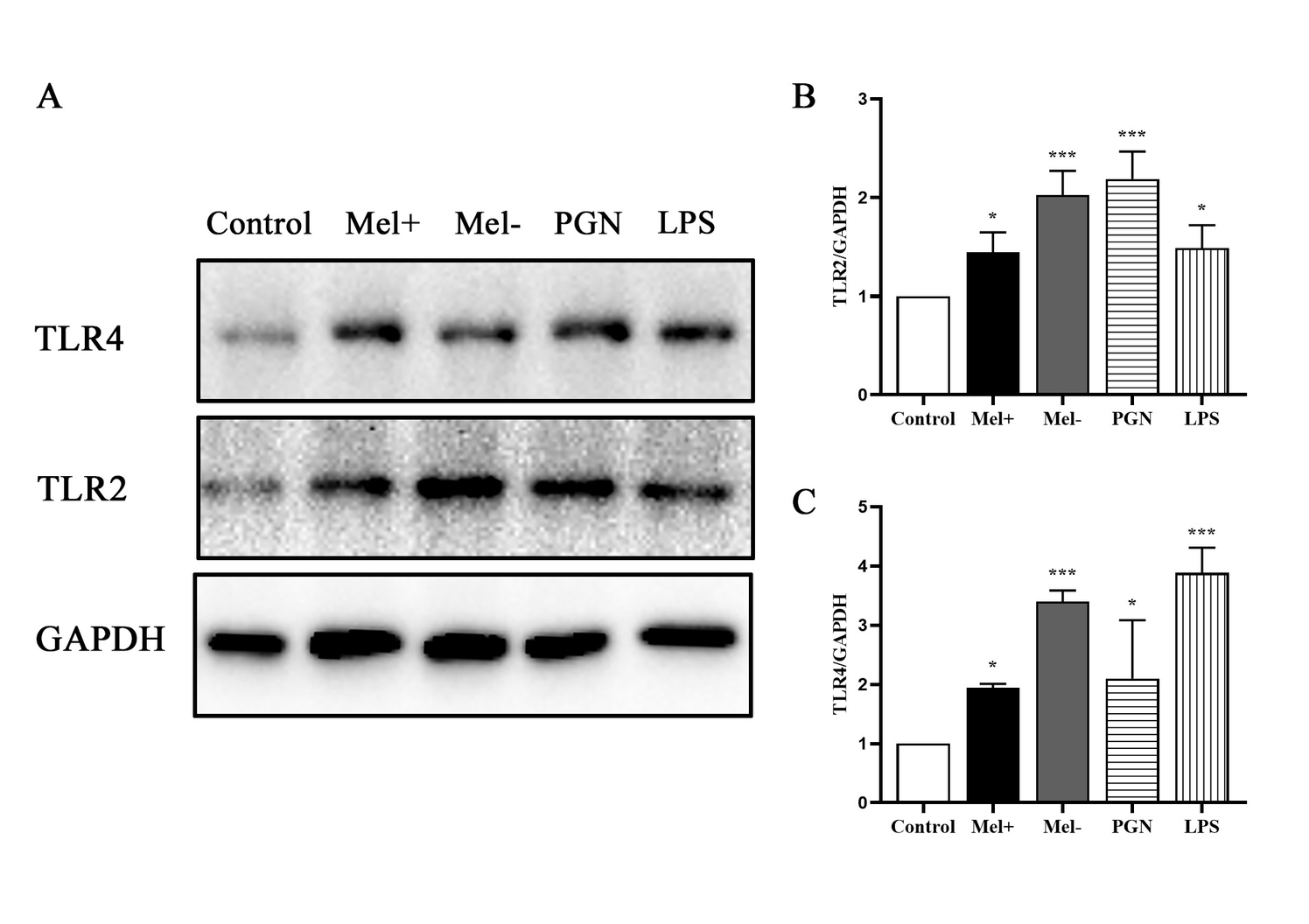

Supplement: S2 Fig — (A) THP-1 macrophages were treated with melanin ghosts (MGs), Mel+ or Mel˗ conidia (MOI = 10), Peptidoglycan (PGN) (20 μg/mL) and LPS (100 ng/mL) for 12 h. The protein levels of TLR2 and TLR4 were analyzed by western blotting. (B, C) The ratios of TLR2/GAPDH and TLR4/GAPDH were calculated. *P < 0.05, **P < 0.01, ***P < 0.001, for one-way ANOVA followed by Bonferroni’s test. The results are representative of three experiments. Representative images are displayed. (TIF) [file pntd.0011281.s002.tif]

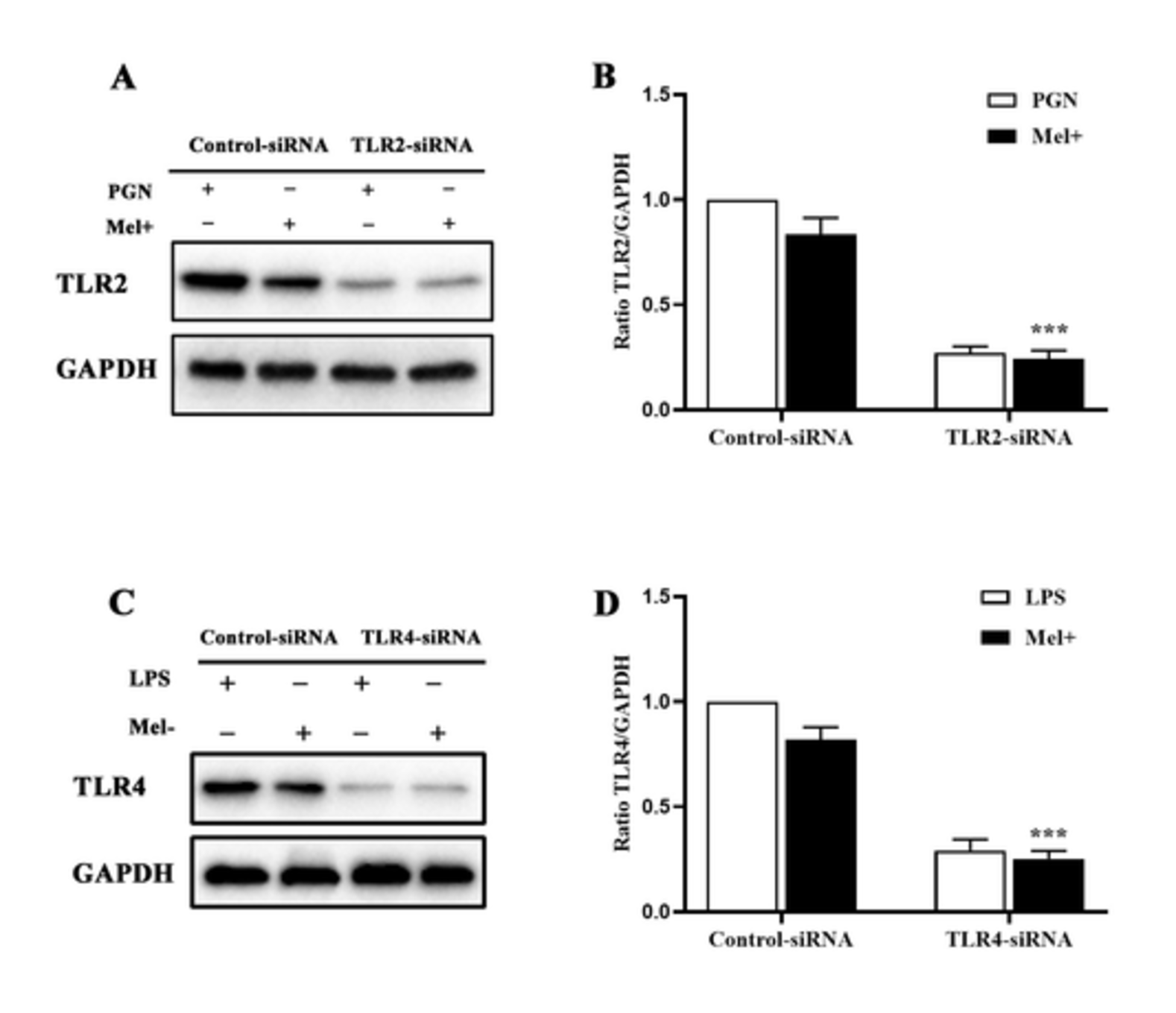

Supplement: S3 Fig — Western blotting of (A) TLR2 and (C) TLR4 in THP-1 macrophages transfected with TLR2-siRNA, TLR4-siRNA or negative control-siRNA, then treated with Mel+ conidia (MOI = 10), PGN (20 μg/mL) and LPS (100 ng/mL) for 12 h. (B, D) The ratios of TLR2/GAPDH and TLR4/GAPDH were calculated. ***P < 0.001, for an unpaired t-test. Data are representative of three experiments and representative images are shown. (TIF) [file pntd.0011281.s003.tif]
