# Supplementary material for: Sporothrix globosa melanin regulates autophagy via the TLR2 signaling pathway in THP-1 macrophages
Source: PLoS Negl Trop Dis. 2023 May 4;17(5):e0011281. doi: 10.1371/journal.pntd.0011281 (PMC10198546; doi:10.1371/journal.pntd.0011281)
Supplement: Attachment — Submitted filename: Autophagy paper Reviews_092622.docx [file pntd.0011281.s004.docx]

**Summary:**

The manuscript, “*Sporothrix globosa* melanin regulates autophagy via the TLR2 signaling pathway in THP-1 macrophages,” describes the induction of autophagy in macrophages, as measured by western blots and microscopy, following infection with *S. globosa.* These findings are interesting and further the understanding of how autophagy is involved in the immune response to fungal infections. Interestingly, the authors find that the induction of autophagy and subsequent effects are increased when the macrophages are infected with a non-melanin producing mutant. These findings add to the growing understanding of the host processes in which fungal melanins interferes, including interaction with TLR2. Overall, I think the authors could help provide clarity to their results with a schematic summarizing their findings (i.e. TLR signaling, autophagy, induction of inflammatory cytokines), and by describing how their findings can be differentiated from induction of inflammatory responses via non-autophagy related phagocytosis or non-autophagy related TLR signaling.

**Questions:**

Do you think the melanin-mediated reduction in autophagy is only related to less activation of TLR2 extracellularly or are there also effects downstream once the conidia are within the macrophage?

Since chloroquine also increases the pH of phagolysosomes (which would presumably contain the conidia), do you think that the reduced levels of cytokine expression in chloroquine-treated cells can be related to impaired phagolysosomal processes in addition to inhibition of autophagolysosomal maturation? Is there a way to look at autophagy-specific downstream effects without also interfering with phagolysosomes (i.e. autophagy specific knockdowns)? I can see that autophagy is induced, and that can lead to immune activation. However, if the “autophagy inhibitors/activators” also interfere with non-autophagy immune signaling in the macrophages, then the degree to which autophagy is specifically responsible for this immune activation is unclear.

Do you think ROS/cytokine production can be triggered by additional immune mechanisms outside of autophagy (i.e. NF-kB signaling, MAP Kinase)? This can be better described or illustrated in a summary figure.

Does the induction of autophagy kill *S. globosa?* Can a survival experiment be done to show whether *S. globosa* survives better in autophagy deficient cells? Do melanized conidia get phagocytosed less than the non-melanized mutant?

Do the conidia associate with the autophagolysosome in the macrophages?

**Introduction:**

General: I think there could be more description of how autophagy participates in antifungal immunity to help emphasize the importance of autophagy, particularly in the paragraph from lines 17-33. Similarly, I think a paragraph describing of LAP (LC3-associated phagocytosis) versus phagocytosis versus typical autophagy would help guide readers in understanding the results.

Lines 34-39 seems awkwardly worded. It might help readers to rephrase or reorder the sentences to flow better.

Lines 47 to 50 can have one or two additional sentences to describe the experiments done in the paper and major findings.

**Materials and Methods:**

Line 63: does “identified,” refer to identification as the *S. globosa* species or as a melanin mutant? I assume species. It would also be good to describe how the melanin-deficient mutant was either found or made in the lab (i.e. random mutagenesis?).

Line 72: Not sure if stimulated is the right word as two are inhibitors.

Section 2.4 should include information about concentration of antibody used and how the western blots were quantified.

Section 2.5 could benefit from explaining what the cutoff/threshold for the puncta size was (i.e. if small puncta were included, if so, how small?). A brief explanation, like the one in lines 145-146 about how this method works would be helpful. Also, it would be good to include exposure information.

Line 102: Briefly state the premise of the assay, such as, “A dichlorofluorescein assay was used to measure ROS, where ROS in the cell oxidize the dye and cause it to fluoresce.”

Section 2.7: Provide information on how the cytokines were collected. From the supernatant I assume?

**Results and Figures:**

It will be helpful to provide a summary diagram at the end of the manuscript to summarize and demonstrate the major findings of the paper and how they all come together. For example, you can show TLR2 signaling to autophagosome maturation to inflammatory cytokine and ROS release and where melanin interferes.

For the bar graphs, it would be beneficial to include the individual data points in the graph, either as a scatter plot alone or overlayed on the bars themselves. The font on these graphs and on the microscopy graphs (Figure 2 and 7) are also small and might be difficult to read without zooming on the image (particularly in Figure 1), so increasing the text size would be good.

The figure legends should include the information regarding which statistical tests were performed, as described in Methods section 2.9. For western blot images and microscopy images, it should be noted that the image is representative. The legend for the microscopy puncta counts should state the number of cells counted, as described in the methods section 2.5.

There are a few instances in which the full figure is only referenced. It would help to reference the specific panels when the data is being discussed to help readers follow better, for example, Section 3.5. Additionally, sometimes the Figure/panels are only referenced once in the paragraph or section, while being discussed throughout, which can be difficult to follow.

Lines 137, 141, 180: Add a sentence indicating what this pattern means.

Section 3.1 does not reference Figure 1E,F.

Figure 3: The western blot does not have a panel attributed to it. It might also help readers to reorder the quantifications to align with the order on the western blot.

Section 3.3/Figure 4: Some of the changes described in this section are more difficult to see than the previous western blot figures. I think quantifying the western blot bands, as in Figure 1, would help strengthen the arguments made in 3.3 by making the differences and patterns clear.

Line 205: I am not sure if mechanisms is the right word.

Line 209: Can remove, “the enhancement of“

Line 212: I don’t think “expression level” is the right word when measuring the direct quantity of ROS and not expression levels of a gene that produces the ROS.

Line 241: Should only reference Supplementary Figure 1?

Line 244: Should reference Supplementary Figure 2?

Figure 6: There are no statistics for Panel D.

Figure 7: It might be better to break the figure into Panels A-D rather than just A and B.

Lines 280-282: Awkward phrasing.

Section 3.6. I think the results in 3.6/Figure 8 can be better explained, particularly explaining the significance of the trends in cytokine levels following TLR2 KD and treatment with chloroquine/rapamycin.

Lines 287-289: I don’t think this statement is entirely correct. It seems that the treatment of chloroquine in the TLR2 KD infected cells did not decrease the levels of proinflammatory cytokines compared to the untreated TLR2 KD infected cells, nor does it seem that rapamycin increase proinflammatory cytokine production in the TLR2 KD infected cells, with the exception of IFN-gamma. Could you provide statistical evidence of this on the graph? Are the control (not TLR2 KD) different from the data in Figure 5?

**Discussion:**

Lines 308-311 can be better explained.

Line 318: I am not sure that you can state that it was protective, as survival outcome of the fungus was not looked at.

Line 319: degradation is repeated.

Line 320: Should it be “induction” instead of “suppression”?

Line 365: Besides reducing the production of ROS melanin is also an antioxidant and shields against ROS that is produced.
