# Supplementary material for: Sporothrix globosa melanin regulates autophagy via the TLR2 signaling pathway in THP-1 macrophages
Source: PLoS Negl Trop Dis. 2023 May 4;17(5):e0011281. doi: 10.1371/journal.pntd.0011281 (PMC10198546; doi:10.1371/journal.pntd.0011281)
Supplement: Attachment — Submitted filename: Response.docx [file pntd.0011281.s005.docx]

**Dear Editors and Reviewers:**

Thank you for your letter and for the reviewers’ comments concerning our manuscript titled “*Sporothrix globosa* melanin regulates autophagy via the TLR2 signaling pathway in THP-1 macrophages” (PNTD-S-22-01514). We have studied the comments carefully and addressed each in turn, with point-by-point revisions highlighted in red font in the revised manuscript. The main corrections to the paper and our responses to the reviewers’ comments are listed below.

**Best regards,**

**Dr. Y Cui**

**Reviewer #1**

**Q1.** *Line 63: does “identified,” refer to identification as the S. globosa species or as a melanin mutant? I assume species. It would also be good to describe how the melanin-deficient mutant was either found or made in the lab (i.e. random mutagenesis?).*

**A1.** The word “identified” refers to identification of the melanin mutant in line 63. Both strains were identified as *S globosa* using traditional morphological and molecular identification methods; the latter involved amplification and sequencing of the calmodulin gene(1). Moreover, the morphological and ultrastructural features of both strains were compared by light microscopy and transmission electron microscopy, respectively. No other structures, expect for the loss of melanin, were altered in the melanin mutant.

An albino mutant strain (Mel˗) of the above-mentioned clinical isolate was constructed by author Dr. Y Song using ultraviolet light at 254 nm as described previously(2), and the strain was stored in our laboratory. This has been explained in the article and the necessary citations regarding the strain have been provided.

**Q2.** *Line 72: Not sure if stimulated is the right word as two are inhibitors.*

**A2.** Considering the reviewer’s suggestion, we have revised the sentence (Lines 71–73) as follows: “Where appropriate, cells were pre-incubated with 20 µM chloroquine, 500 nM wortmannin or 5 µM rapamycin (all from MedChemExpress, Shanghai, China) for different time periods.”

**Q3.** *Section 2.4 should include information about concentration of antibody used and how the western blots were quantified.*

**A3.** The text has been modified accordingly. “Total protein was collected from cells using RIPA Lysis Buffer (Beyotime, Suzhou, China). Whole protein extracts were separated by 12% sodium dodecyl sulfate-polyacrylamide gel electrophoresis and transferred onto polyvinylidene difluoride membranes (Millipore Corporation, Temecula, CA, USA). Western blotting was performed according to the manufacturer’s instructions using the following primary antibodies: rabbit anti-LC3A/B at 1:1000 (Cat. #12741, Cell Signaling Technology, Beverly, MA, USA), rabbit anti-Beclin1 at 1:1000 (Cat. #3495, Cell Signaling Technology), rabbit anti-SQSTM1/p62 at 1:1000 (Cat. #8025, Cell Signaling Technology), mouse anti-GAPDH antibody at 1:4000 (Cat. # T0004, Affinity Biosciences, Cincinnati, OH, USA), rabbit anti-TLR2 antibody at 1:1000 (Cat. # 12276, Cell Signaling Technology), rabbit anti-TLR4 antibody (Cat. # BS3489, Bioworld Technology, MN, USA) at 1:500 and rabbit anti-Atg7 antibody at 1:1000 (Cat. # DF6130, Affinity Biosciences). After washing three times in TBST solution, the membranes were incubated with the respective secondary antibodies (anti-mouse HRP or anti-rabbit HRP at 1:4000, Affinity Biosciences) for 1 h at room temperature. The blots were assessed by enhanced chemiluminescence detection (Affinity Biosciences). The mean pixel density of each protein band was quantified using Image J (NIH Image J system, 1.8.0). Anti-GAPDH expression was analyzed as an internal control, and protein expression was quantified as the ratio of the specific band to GAPDH.”

**Q4.** *Section 2.5 could benefit from explaining what the cutoff/threshold for the puncta size was (i.e. if small puncta were included, if so, how small?). A brief explanation, like the one in lines 145-146 about how this method works would be helpful. Also, it would be good to include exposure information.*

**A4.** The experiment was performed according to previous literature(3, 4) and the manufacturer’s instructions. Magnified images of the dots are shown in the new Fig. 1 and the circled dots were counted, in accordance with previously published methods(5). The number of dots per cell was obtained by dividing the total number of dots by the number of cells in each microscopic field (see new Fig. 1 below). We also added a brief explanation of the method to lines 145–146 in the revised manuscript.

**Q5.** *Briefly state the premise of the assay, such as, “A dichlorofluorescein assay was used to measure ROS, where ROS in the cell oxidize the dye and cause it to fluoresce.”*

**A5.** We have added more detail as requested and modified the revised manuscript as follows:

“A dichlorofluorescein assay was used to measure ROS (32). The principal component of this assay is 2,7-dichlorodihydrofluorescein diacetate (DCFH-DA), which is easily oxidized to fluorescent dichlorofluorescein (DCF) by intracellular ROS, thereby enabling the quantification of ROS levels. Briefly, THP-1 macrophages (2 × 10^5^/well) were plated into 96-well black round-bottom plates (Corning, New York, NY, USA) and infected with *S. globosa* (Mel+ or Mel˗) conidia (MOI = 10). After a 12-h incubation, 2 DCFH-DA (Beyotime) was added to each well to a final concentration of 10 µmol/L, and the cells were incubated at 37°C in a dark room for 20 min. The results were obtained using a fluorescence plate reader at 488 nm for excitation and 535 nm for emission. The test was performed in triplicate.”

**Q6.** *Section 2.7: Provide information on how the cytokines were collected. From the supernatant I assume?*

**A6.** Yes, the cytokines were collected from the supernatant. We have edited the relevant section of the revised manuscript to clarify this.

Q7. It will be helpful to provide a summary diagram at the end of the manuscript to summarize and demonstrate the major findings of the paper and how they all come together. For example, you can show TLR2 signaling to autophagosome maturation to inflammatory cytokine and ROS release and where melanin interferes.

**A7.** We have added a summary diagram shown in Figure 11 and edited the main text accordingly.

Q8. For the bar graphs, it would be beneficial to include the individual data points in the graph, either as a scatter plot alone or overlayed on the bars themselves. The font on these graphs and on the microscopy graphs (Figure 2 and 7) are also small and might be difficult to read without zooming on the image (particularly in Figure 1), so increasing the text size would be good.

**A8.** According to the reviewer's comment, we had changed the figure to a bar graph superimposed with a scatter plot. As is shown in the new Fig 2 below, scatter plots are less aesthetic than bar graphs as there were too few scatters. Therefore, data are represented as column plots in the manuscript. The font presented in this figure was also modified as requested.

Q9. The figure legends should include the information regarding which statistical tests were performed, as described in Methods section 2.9. For western blot images and microscopy images, it should be noted that the image is representative. The legend for the microscopy puncta counts should state the number of cells counted, as described in the methods section 2.5.

**A9.** The figure legends have been modified according to the reviewer's comments.

Q10. There are a few instances in which the full figure is only referenced. It would help to reference the specific panels when the data is being discussed to help readers follow better, for example, Section 3.5. Additionally, sometimes the Figure/panels are only referenced once in the paragraph or section, while being discussed throughout, which can be difficult to follow.

**A10.** Specific figure panels are now referenced as appropriate in the text, as suggested by the reviewer.

Q11. Generally, the results are clearly presented and the figures have sufficient clarity. I think it may be hard to follow the story that is being told at times, so I think including a summary figure would be beneficial in ensuring the readers are on the same page by the end of the results section.

**A11.** We have added a summary diagram to clarify our findings.

Q12. Lines 137, 141, 180: Add a sentence indicating what this pattern means.

**A12.** Mel+ conidia represent the wild-type strain of *S. globosa* (Mel+, FHJU12082703), and Mel˗ conidia represent a melanin-deficient mutant.

Q13. Section 3.1 does not reference Figure 1E, F.

**A13.** These figure panels are now cited in the text.

Q14. Figure 3: The western blot does not have a panel attributed to it. It might also help readers to reorder the quantifications to align with the order on the western blot.

**A14.** The figures have been adjusted as requested.

Q15. Section 3.3/Figure 4: Some of the changes described in this section are more difficult to see than the previous western blot figures. I think quantifying the western blot bands, as in Figure 1, would help strengthen the arguments made in 3.3 by making the differences and patterns clear.

**A15.** Western blot bands have been quantified as requested.

Q16. Line 205: I am not sure if mechanisms is the right word.

**A16.** The word “mechanisms” has been replaced with “effect”.

Q17. Line 209: Can remove, “the enhancement of”.

**A17.** This phrase has been deleted.

Q18. Line 212: I don’t think “expression level” is the right word when measuring the direct quantity of ROS and not expression levels of a gene that produces the ROS.

**A18.** We have replaced “expression level” with “production”.

Q19. Line 241: Should only reference Supplementary Figure 1?

**A19.** This has been corrected.

Q20. Should reference Supplementary Figure 2?

**A20.** This has been corrected.

Q21. Figure 6: There are no statistics for Panel D.

**A21.** The original Figure 6 is now Figure 8 in the revised manuscript and NS denotes no significant difference, which has been labeled in Fig. 8D.

Q22. Figure 7: It might be better to break the figure into Panels A-D rather than just A and B.

**A22.** The original Figure 7 is now Figure 9 in the revised manuscript and has been split into panels A–D.

Q23. Lines 280-282: Awkward phrasing.

**A23.** This sentence has been rewritten.

Q24. The general premises of the manuscript that 1. *S. globosa* conidia induces autophagy and immune response mediated by TLR2, and 2. melanized conidia induce less autophagy and induce less cytokine/immune response, are supported by the data presented. I think the limitations (discussion of the non-autophagy related pathways that can activate the immune response, and discussion of the non-autophagy immune pathways that the "autophagy activators or inhibitors" can interfere with) are not discussed as extensively as they can, especially when interpreting the data.

**A24.** An explanation has been added to the Discussion section, as requested, and the changes are indicated in red font. It is known that TNF-α and IL-6, as critical inflammatory mediators, help resist fungal infection and promote the infiltration of inflammatory cells to remove pathogens, such as increasing the killing of phagocytosed *S. schenckii* (7). Consistent with this, we previously showed that expression of the proinflammatory cytokines TNF-α and IL-6 increased during the phagocytosis of *S. globosa* by macrophages(8). The present study found that TNF-α, IL-6, IL-1β and IFN-γ were all induced in THP-1 macrophages infected with *S. globosa* conidia, and that three of these proinflammatory cytokines (TNF-α, IL-6 and IFN-γ) were positively related to autophagy in THP-1 macrophages. The levels of IL-1β were negatively associated with autophagic activity, indicating that the cytokines induced by *S. globosa* in THP-1 macrophages may be regulated by autophagy to produce a stronger host immune response. In addition, since chloroquine could also increase the pH of phagolysosomes (which would presumably contain the conidia), the reduced levels of cytokine expression in chloroquine-treated cells may be related to impaired phagolysosomal processes, in addition to the inhibition of autophagolysosomal maturation. This confirmed that the changes in cytokines were a consequence of autophagy.

Q25. I think the results in 3.6/Figure 8 can be better explained, particularly explaining the significance of the trends in cytokine levels following TLR2 KD and treatment with chloroquine/rapamycin.

**A25.** An explanation has been added to the Discussion section and the changes are indicated in red font. It has been reported that TLR2 signals primarily induce inflammatory responses through the MyD88-dependent pathway(9), which may also be the signaling pathway of autophagy regulators to control inflammation, resulting in insignificant changes in TLR2 knockdown cells. Carlos and colleagues revealed that spleen cells in TLR2^˗/˗^ mice showed no release, or decreased production of, proinflammatory cytokines such as IFN-γ and IL-6 after infection with sporotrichosis(10). We propose that autophagy regulators, such as chloroquine, only influence events downstream of autophagosomes and might not be affected by TLR2, resulting in no clear trend in cytokine production.

Q26. Lines 287-289: I don’t think this statement is entirely correct. It seems that the treatment of chloroquine in the TLR2 KD infected cells did not decrease the levels of proinflammatory cytokines compared to the untreated TLR2 KD infected cells, nor does it seem that rapamycin increase proinflammatory cytokine production in the TLR2 KD infected cells, with the exception of IFN-gamma. Could you provide statistical evidence of this on the graph? Are the control (not TLR2 KD) different from the data in Figure 5?

**A26.** We compared the mean levels of proinflammatory cytokine production in TLR2-knockdown infected cells with those in chloroquine- or rapamycin-treated TLR2-knockdown infected cells and no inhibitory or facilitatory effect on proinflammatory cytokine production was observed in the treated cells. Therefore, as suggested by the reviewer, we removed the related statements from the text.

Q27. Lines 308-311 can be better explained.

**A27.** We have rewritten this section of the text.

Q28. Line 318: I am not sure that you can state that it was protective, as survival outcome of the fungus was not looked at.

**A28.** We have changed the word “protective” to “promoting”.

Q29. Line 319: degradation is repeated.

**A29.** The repeated word has been deleted.

Q30. Line 320: Should it be “induction” instead of “suppression”?

**A30.** We agree and have changed “suppression” to “induction”.

Q31. Line 365: Besides reducing the production of ROS melanin is also an antioxidant and shields against ROS that is produced.

**A31.** We have added an explanation that melanin is also an antioxidant that shields against ROS.

Q32. Do you think the melanin-mediated reduction in autophagy is only related to less activation of TLR2 extracellularly or are there also effects downstream once the conidia are within the macrophage?

**A32.** Our results uncovered novel roles of TLR2 in regulating *S. globosa*-induced autophagy in macrophages, and *S. globosa* melanin could inhibit macrophage functionality by resisting macrophage autophagy through the regulation of TLR2 expression. Similar results were found in our previous study. We believe that the mechanism may be based on the absence of melanin leading to partial exposure of the antigen.

We further explored the function of the TLR2/MyD88 signaling pathway during *S. globosa* infection of macrophages and our results revealed that the expression of MyD88 was elevated in *S. globosa*-infected macrophages (see new Fig. 3 below). This indicated that MyD88 may operate downstream of the TLR2 signaling pathway. Our results are preliminary and need to be confirmed in further experiments.

Q33. Since chloroquine also increases the pH of phagolysosomes (which would presumably contain the conidia), do you think that the reduced levels of cytokine expression in chloroquine-treated cells can be related to impaired phagolysosomal processes in addition to inhibition of autophagolysosomal maturation? Is there a way to look at autophagy-specific downstream effects without also interfering with phagolysosomes (i.e. autophagy specific knockdowns)? I can see that autophagy is induced, and that can lead to immune activation. However, if the “autophagy inhibitors/activators” also interfere with non-autophagy immune signaling in the macrophages, then the degree to which autophagy is specifically responsible for this immune activation is unclear.

**A33.** According to the reviewer's suggestion, we designed an experiment using a specific Atg7-siRNA to knockdown the expression of Atg7. Atg7 encodes an E1-like activating enzyme that is necessary for autophagy and is a critical component in maintaining the function of the microtubule‐associated protein light chain 3 (LC3) conjugation system and the ATG12 conjugation system. Loss of Atg7 disables the formation of double-membrane autophagosomes, resulting in the inability to initiate autophagy(11). The proinflammatory factors (TNF-α, IL-6 and IFN-γ) in THP-1 macrophages transfected with Atg7-siRNA were significantly decreased compared with cells transfected with the Ctrl-siRNA (*P* < 0.05), whereas IL-1β showed an upward trend. This result confirmed that the S. globosa-induced autophagic response was interfering with the expression of autophagy-specific genes downstream . These results are presented and described in the revised manuscript.

Q34. Do you think ROS/cytokine production can be triggered by additional immune mechanisms outside of autophagy (i.e. NF-kB signaling, MAP Kinase)? This can be better described or illustrated in a summary figure.

**A34.** We have added a summary diagram, which is cited in the main text.

Q35. Does the induction of autophagy kill *S. globosa*? Can a survival experiment be done to show whether *S. globosa* survives better in autophagy deficient cells?

**A35.** After considering the reviewer’s comments, we designed an experiment as follows. A fungal killing assay was conducted in THP-1 macrophages pre-treated with or without chloroquine (20 µM) and rapamycin (5 µM) after harvesting the Mel+ and Mel˗ strains. We found that Mel˗ conidia had lower survival rates than Mel+ conidia, and the autophagy modulators had little effect. Furthermore, higher numbers of conidia CFUs were recorded in the chloroquine group (*P* = 0.274) compared with the rapamycin group (*P* = 0.210), although the standardized mean difference was not statistically significant. Moreover, statistically significant differences were detected between the chloroquine and rapamycin groups regardless of the presence of melanin (*P* = 0.035 for Mel+; *P* = 0.041 for Mel˗). We thus consider that autophagy may promote the clearance of *S. globosa* in THP-1 macrophages (see new Fig. 4 below). Further experiments will be necessary to address this issue fully in future studies. (Supplementary Notes: The number of colonies in the control group was diluted prior to calculation, whereas the number of colonies presented in the figure was undiluted to highlight the differences between groups).

Q36. Do melanized conidia get phagocytosed less than the non-melanized mutant?

**A36.** Yes. Our previous study(8) on phagocytosis of *S. globosa* conidia by THP-1 macrophages revealed that the phagocytosis index of *S. globosa* by THP-1 macrophages was enhanced in the absence of melanin (see new Fig. 5 below).

Q37. Do the conidia associate with the autophagolysosome in the macrophages?

**A37.** This is an interesting question. Although our experiments do not include the colocalization of conidia and autophagolysosomes, a previous study reported that *Listeria monocytogenes* could be sequestered by autophagosomes (newly-formed double-membraned organelles), which then fuse with lysosomes, forming an autolysosome in which the content is degraded(12). There is currently no literature on whether conidia are sequestered by autophagosomes; however, this is an interesting question that warrants experimental verification in the future.

**Reviewer #2**

**Q1.** *The major flaw of the experimental design is the prolonged interaction times of up to 24 hours. It is unlikely that after this time conidia have not germinated and started to produce hypha (our calculations of* *conidia germination for this species are about 13-15 h), so the results at late times may be due to conidia germination instead of the suggested kinetic response. In the same line, what are the germination time and rate for both fungal strains, it is assumed it is the same, but this might be wrong. The authors have to demonstrate that both strains show similar germination rates. To minimize the potential effect of hypha production, the authors should perform the experiments with UV-killed cells; in this case, the cell wall is intact and viability lost. A good extra control that the authors should include in the experimental design is yeast-like cells, which do not synthesize melanin in the used experimental conditions. This will reinforce the role of melanin in this host-fungus interaction.*

**A1.** We are grateful for these valuable comments. We regret that we did not consider the influence of hypha production on our experimental results. However, after considering the reviewer’s recommendations, inactivated strains were re-examined in THP-1 macrophages to evaluate the effect on autophagy. Our results showed that there were no changes in the expression of autophagy-related proteins after a 12-h incubation. On the one hand, autophagy-related proteins were assessed at only one time point and the MOI was determined three times because of time constraints. The findings suggested that the cell wall component of *S. globosa* alone may not be sufficient to activate THP-1 macrophage autophagy via the TLR2 signaling pathway. On the other hand, we found that cell autophagy gradually increased with prolonged infection time (between 8 h and 12 h) before the occurrence of germinating hyphae (12 h). Therefore, we propose that, in this context, autophagy is a dynamic process that occurs over a prolonged time period. The main text and Fig. 6 have been revised accordingly (see new Fig. 6 below).

Based on the reviewer’s suggestion, we also introduced yeast cells of *S. globosa* (Mel+, FHJU12082703) into our experiments. First, to check for the levels of autophagy marker proteins of yeast cells, the proteins LC3-II/I, Beclin1 and p62 were tested after exposure to yeast cells or rapamycin (5 μM) at the same time points and MOIs described in the preceding text. Consistent with the results for Mel+ and Mel˗, the expression of autophagy-associated proteins LC3-II/I, p62 and Beclin1 was dependent on incubation time and concentration. In addition, to further explore the function of melanin during autophagy induction by *S. globosa* in THP-1 macrophages, we compared the effects of Mel+ conidia and yeast cells on the induction of autophagy in THP-1 macrophages. The results in yeast cells were consistent with those in the ultraviolet-induced mutant (Mel˗) group, with the expression of autophagy-related markers being more pronounced in the absence of melanin. These new data have been added to the revised manuscript.

**Q2.** *There are different compounds that the authors added to the host-fungus interactions and it is not considered the possibility that this may contain bacterial LPS. The authors should demonstrate that these compounds are LPS-free.*

**A2.** We thank the reviewer for raising this point. All experimental manipulations were performed under sterile conditions and all of the compounds used to analyze host–fungus interactions were sterile reagents. We have added this information to the Materials and Methods section of the main text.

**Q3.** *It is not clear why the authors only focused on TLR2 and TLR4, this is such a narrow repertoire of PRR involved in the interaction with the host and in mediating autophagy.*

**A3.** We agree that this is not the full picture. In response, changes in the mRNA expression levels of Dectin-1, mannose receptor (MR), TLR2 and TLR4 in THP-1 macrophages co-incubated with *S. globosa* conidia were assessed by quantitative PCR (qPCR) analysis. The primer sequences used for qPCR are provided in Table 1. Although increasing mRNA expression levels were observed for all of the PRRs, this difference only reached significance for TLR2 and TLR4 (see new Fig. 7 below). In our previous study, qPCR analysis of the skin lesions of patients with sporotrichosis revealed significantly higher expression levels of TLR2 and TLR4 in the skin lesions compared with the healthy controls. However, this was not the case for Dectin-1 or MR (unpublished), suggesting that TLR2 and TLR4 may be the critical recognition receptors in sporotrichosis. In parallel, we also analyzed transcriptome changes in the keratinocytes infected with *S. globosa*, and among PPRs, only the TLR2 signaling pathway was found to be significantly altered. In addition, in our previous study, we found the receptors TLR2 and TLR4 play an important role during the phagocytosis of *S. globosa* in THP-1 macrophages(8). We therefore have evidence that TLR2 is important in *S. globosa* infection, which may be attributed to the characteristics of the cell wall components in *S. globosa*.

**Q4.** I do not think the parametric analyses used in the statistic section are the most appropriate ones for this study, non-parametric analyses should be included instead.

**A4.** We re-analyzed the data using non-parametric methods and have rewritten the relevant section in the text.

**Q5.** The English usage is acceptable but has room for improvement.

**A5.** We employed a professional editing company to improve the readability of our manuscript.

**References**

1.	Moussa TAA, Kadasa NMS, Al Zahrani HS, Ahmed SA, Feng P, Gerrits van den Ende AHG, et al. Origin and distribution of Sporothrix globosa causing sapronoses in Asia. J Med Microbiol. 2017;66(5):560-9.

2.	Romero-Martinez R, Wheeler M, Guerrero-Plata A, Rico G, Torres-Guerrero H. Biosynthesis and functions of melanin in Sporothrix schenckii. Infect Immun. 2000;68(6):3696-703.

3.	Wu B, Yu L, Wang Y, Wang H, Li C, Yin Y, et al. Aldehyde dehydrogenase 2 activation in aged heart improves the autophagy by reducing the carbonyl modification on SIRT1. Oncotarget. 2016;7(3):2175-88.

4.	Hariharan N, Zhai P, Sadoshima J. Oxidative stress stimulates autophagic flux during ischemia/reperfusion. Antioxid Redox Signal. 2011;14(11):2179-90.

5.	Liu J, Li QX, Wang XJ, Zhang C, Duan YQ, Wang ZY, et al. β-Arrestins promote podocyte injury by inhibition of autophagy in diabetic nephropathy. Cell Death Dis. 2016;7(4):e2183.

6.	Wang H, Joseph JA. Quantifying cellular oxidative stress by dichlorofluorescein assay using microplate reader. Free Radic Biol Med. 1999;27(5-6):612-6.

7.	Drummond RA, Gaffen SL, Hise AG, Brown GD. Innate Defense against Fungal Pathogens. Cold Spring Harb Perspect Med. 2014;5(6).

8.	Guan MQ, Yao L, Zhen Y, Song Y, Cui Y, Li SS. Melanin of Sporothrix globosa affects the function of THP-1 macrophages and modulates the expression of TLR2 and TLR4. Microb Pathog. 2021;159:105158.

9.	Akira S, Takeda K. Toll-like receptor signalling. Nat Rev Immunol. 2004;4(7):499-511.

10.	de C Negrini T, Ferreira LS, Arthur RA, Alegranci P, Placeres MCP, Spolidorio LC, et al. Influence of TLR-2 in the immune response in the infection induced by fungus Sporothrix schenckii. Immunol Invest. 2014;43(4):370-90.

11.	Mizushima N, Levine B, Cuervo AM, Klionsky DJ. Autophagy fights disease through cellular self-digestion. Nature. 2008;451(7182):1069-75.

12.	Anand PK, Tait SW, Lamkanfi M, Amer AO, Nunez G, Pagès G, et al. TLR2 and RIP2 pathways mediate autophagy of Listeria monocytogenes via extracellular signal-regulated kinase (ERK) activation. J Biol Chem. 2011;286(50):42981-91.

Fig 1. Representative images of LC3 staining, as determined by measurement of the fluorescent intensity in cells (A: THP-1 macrophages; B: podocytes (5)) in different groups infected with RFP–GFP–LC3 adenovirus for different time periods.

Fig 2. Calculated ratios of the mean grayscale intensity of bands corresponding to LC3II and GAPDH, with bar graphs superimposed with scatter plots. ***P* < 0.01 and ****P* < 0.001.

Fig 3. Western blotting analysis of (A) MyD88 in THP-1 macrophages treated with Mel+ conidia (MOI = 10) for 12 h. (B) The ratio of MyD88/GAPDH, as determined by grayscale analysis. **P* < 0.05, unpaired t-test. Data are representative of three experiments and representative images are shown.

Fig 4. Killing assay of *S. globosa* conidia in activated THP-1 macrophage autophagy. (A) Colonies of the Mel+ (a–d) and Mel˗ (e–h) strains pretreated with or without chloroquine (20 µM, 4 h) or rapamycin (5 µM, 12 h) in THP-1 macrophages for 12 h, and then subcultured on Sabouraud dextrose agar at 28°C for 7 days. (B) Strain counts were expressed as the Log CFU/mL. Bars represent the means ± SD of three independent samples. **P* < 0.05 and ****P* < 0.001, for one-way ANOVA followed by Bonferroni’s post-test; ^#^ *P* < 0.05, for the unpaired t-test.

Fig 5. Confocal microscopy showing the phagocytosis of *S. globosa* conidia by THP-1 macrophages. (A) Representative images of phagocytosis of S. *globosa* conidia by THP-1 macrophages under a laser scanning confocal microscope at a magnification of 1800× (bar = 20 μm), showing cells infected by Mel+ or Mel˗ conidia. From left to right, fluorescence image showing the DAPI-labeled cell nucleus (blue), fluorescence image showing the TRITC-labeled THP-1 macrophages (red), fluorescence image showing the FITC-labeled Mel+ or Mel˗ conidia of *S. globosa* (green), and a merge overlay of all images. (B) Decreased THP-1 macrophage phagocytosis of Mel+ conidia compared with cells infected with Mel˗ conidia. The phagocytosis index was measured as the percentage of THP-1 macrophages with internalized conidia multiplied by the average number of conidia per THP-1 macrophage. **P* < 0.05. The values are expressed as the mean ± SD from triplicate determinations of a representative experiment(8).

Fig 6. Effect of inactivated *S. globosa* (Mel+ and Mel˗) on THP-1 macrophages. (A) Western blot analysis of THP-1 macrophages pretreated with or without rapamycin (5 µM, 12 h), and subsequently infected with inactivated Mel+ or Mel˗ *S. globosa* conidia (MOI = 10) for 12 h. (B–D) The ratios of LC3-II/GAPDH, Beclin1/GAPDH and p62/GAPDH were calculated; **P* < 0.005, ***P* < 0.01; *^#^P* < 0.05, ^##^*P* < 0.01, for one-way ANOVA followed by Bonferroni’s post-test. Representative images are shown.

Fig 7. Real-time PCR analysis of the mRNA levels of TLR2, TLR4, MR and Dectin-1 for THP-1 macrophages treated with Mel+ conidia (MOI = 10) for 12 h. ***P* < 0.01, ****P* < 0.001.

Table 1 qPCR primer sequences

| Gene | Forward primers | Reverse primers |
| --- | --- | --- |
| GAPDH | CTGCACCACCAACTGCTTAG | GTCTGGGATGGAAATTGTGA |
| MR | GGGTTGCTATCACTCTCTATGC | TTTCTTGTCTGTTGCCGTAGTT |
| Dectin-1 | GGAAGCAACACATTGGAGAATGG | AGAACCCCTGTGGTTTTGACA |
| TLR2 | TCCGTCTTTTTGATGAGAACAATG | ACTCCAGGTAGGTCTTGGTGTTCA |
| TLR4 | GGTGAGTAATTCCATGGTGCACTA | TTCCCTCTGCACTGGAAGCT |
