# Supplementary material for: Sporothrix globosa melanin regulates autophagy via the TLR2 signaling pathway in THP-1 macrophages
Source: PLoS Negl Trop Dis. 2023 May 4;17(5):e0011281. doi: 10.1371/journal.pntd.0011281 (PMC10198546; doi:10.1371/journal.pntd.0011281)
Supplement: Attachment — Submitted filename: response 2.docx [file pntd.0011281.s006.docx]

**Dear Editors and Reviewers:**

Thank you for your letter and for the reviewers’ comments concerning our manuscript titled “*Sporothrix globosa* melanin regulates autophagy via the TLR2 signaling pathway in THP-1 macrophages” (PNTD-S-22-01514). We have studied the comments carefully and addressed each in turn, with point-by-point revisions highlighted in red font in the revised manuscript. The main corrections to the paper and our responses to the reviewers’ comments are listed below.

**Best regards,**

**Dr. Y Cui**

**Reviewer #1**

**Q1.** *Figure 1 should probably be two separate figures.
I find the legend for Figure 1 particularly unclear, and the legend makes it sounds like it is the same experiment in both halves even though it is not.
I think having better/larger labelling of Figure 1 will help with understanding. (I.e. if “Beclin”,”LC3-II”, etc were written above the relevant panels it will help in understanding what each column is quantifying). It might help to also label each row (I.e. write “Mel+” next to A and B). Also, it is important to label the X-axis to understand the graph better.
The numbers and labelling of figures can generally be larger, but especially for Figure 1.
Please reference specific panels in the text. For example, one reference to “Figure 5” should be split up into references to the individual panels in the discussion of the findings, when mentioned, throughout the rest of the paragraph.*

**A1.** Thank you for your valuable comment. We have made the necessary changes to the figures and labelling to ensure clarity. And we have also made sure to reference the individual panels in the text when discussing the findings.

**Q2.** *Similarly, Figure 7 should be split into references to individual panels throughout the paragraph rather than just referencing the entire figure in the final sentence of the paragraph. Currently that paragraph mostly seems in reference to the supplementary material rather than actual data presented in Figure 7 which only seems mentioned as an afterthought. Reference to 9B,D comes before 9A,C.*

**A2.** According to the reviewer's suggestion, Figure 7 has been split into references to individual panels throughout the paragraph. Moreover, as suggested by the reviewer we adjusted the reference in the text.

**Q3.** *The GADPH Western Blot bands in Supplementary Figure 2A look more pixelated, geometrical, and darker than the rest of the bands presented in the manuscript. Were these the result of overexposure or a processing difference? If the bands were manipulated in a way, please make sure to mention that in the manuscript.*

**A3.** Thank you for bringing this to our attention. We have investigated the issue and have determined that the GAPDH Western Blot bands in Supplementary Figure 2A were not manipulated in any way. They appear darker and more pixelated due to a difference in the exposure time of the Western Blot.

**Q4.** *Quantification data and unprocessed western blot image files do not seem to be available in the supplementary material.*

**A4.** Quantification data and unprocessed western blot image files have been provided in the supplementary material.

**Reviewer #2**

**Q1.** *The authors addressed most of my concerns, and I am satisfied with the replies and new data, which make the whole dataset stronger. However, I still have a concern related to the potential contamination with LPS (Q2. There are different compounds that the authors added to the host-fungus interactions and it is not considered the possibility that this may contain bacterial LPS. The authors should demonstrate that these compounds are LPS-free).
I have no doubts about the authors' aseptic technique. I am sure they are at the highest standards. However, the compounds used to interact with immune cells are often produced by genetics engineering using bacteria as a host, and the purification procedures may not get rid of LPS. Thus, I still think the authors should address this potential handicap in the experimental design.*

**A1.** We thank the reviewer for raising this point. All reagents, such as RPMI 1640 medium, chloroquine, wortmannin, rapamycin and Hiperfect transfection reagent et al., were free of LPS as assessed by the *Limulus* amebocyte lysate kit from Bioendo (Xiamen Bioendo Technology, Xiamen, China) (<0.1EU/ml, below detection limit*).* And we have added this information to the Materials and Methods section of the main text. Furthermore, in order to validate the experimental results, cytokine release analysis was re-assessed in THP-1 macrophages treated with the stimulus as described(1) above after pre-incubation with 5μg/mL polymyxin B (Solarbio, Beijing, China) after harvesting the Mel+ conidia. As shown in new Figure1 below, no differences were observed in the polymyxin B treatment groups and those groups not treated with polymyxin B, in which the results were not disturbed by the LPS.

Fig 1. Proinflammatory cytokines released by macrophages treated with or without polymyxin B following exposure to *S. globosa*. THP-1 macrophages pretreated with or without polymyxin B and subsequently infected with *S. globosa* (Mel+) conidia (MOI = 10) for 12 h. Concentrations of inflammatory cytokines IL-6 (A), IL-1β (B), IFN-γ (C) and TNF-α (D) in the THP-1 macrophage groups.

# References

1.	Martínez-Álvarez JA, Pérez-García LA, Mellado-Mojica E, López MG, Martínez-Duncker I, Lópes-Bezerra LM, et al. Sporothrix schenckii sensu stricto and Sporothrix brasiliensis Are Differentially Recognized by Human Peripheral Blood Mononuclear Cells. Front Microbiol. 2017;8:843.
